# Obituary

**Published:** 1893-04

**Authors:** 


### Obituary.

---

DR. LAURENCE JOHNSON died in New York, March 18, 1893, after a short illness from pneumonia. He was born June 7, 1845, in South Butler, Wayne county, N. Y., and in 1863 enlisted as a private in the Ninth New York Heavy Artillery. In 1864, he was appointed First Lieutenant and was attached to the Eighth United States Colored Artillery at Paducah, Ky., and later on the Potomac. Following the close of the war he studied medicine and received his degree from Bellevue Hospital Medical College in 1868. He was President of the Medical Society of the County of New York, a member of the board of managers of the New York Physicians' Mutual Aid Association, of the New York Society for the Relief of Widows and Orphans of Medical Men, of the Medical Society of the State of New York, and of the New York Academy of Medicine, of which he was at one time librarian, and at the time of his death a trustee. He was also a member of the Academy of Sciences and of the Torrey Botanical Club, the Military Order of the Loyal

Legion, of the Grand Army of the Republic, etc. He had for several years been lecturer on Medical Botany and Clinical Medicine in the University of the City of New York, and visiting physician to the Randall's Island Hospital, as well as having connection with the medical staff of other public hospitals and dispensaries. In 1880–90, he was one of the "Committee of Revision and Publication of the Pharmacopeia of the United States of America," and was the author of works on medical formulas and on the medical botany of North America.

